# Evaluation of Role of Myofibroblasts in Oral Cancer: A Systematic Review

**DOI:** 10.5005/jp-journals-10005-1370

**Published:** 2016-09-27

**Authors:** Harjeet K Sekhon, Keya Sircar, Gurbani Kaur, Muneet Marwah

**Affiliations:** 1 Senior Lecturer, Department of Oral Pathology and Microbiology, D.J. College of Dental Sciences & Research, Modinagar, Uttar Pradesh, India; 2 Head, Department of Oral Pathology and Microbiology, Faculty of Dentistry, Jamia Millia Islamia, New Delhi, India; 3 Ex-post Graduate Student, Department of Periodontology, Dr. D.Y. Patil Dental College and Hospital, Dr. D.Y. Patil Vidyapeeth, Pune, Maharashtra, India; 4 Postgraduate, Department of Prosthodontics, Government Dental College Thiruvananthapuram, Kerala, India

**Keywords:** Myofibroblast, Oral cancer, Precancer.

## Abstract

**Aim:**

To conduct a systematic review on the role of myofibroblasts in progression of oral cancer. The myofibroblast is essential for the integrity of the mammalian body by virtue of its role in wound healing, but it also plays a negative role due to their role in promoting tumor development.

**Settings and design:**

Systematic review.

**Materials and methods:**

Bibliographic searches were conducted in several electronic databases using all publications in PubMed, PubMed central, EMBASE, CancerLit, Google scholar, and Cochrane CCTR between 1990 and June 2015.

**Results:**

The search of all publications from various electronic databases revealed 1,371 citations. The total number of studies considered for systematic review was 43. The total number of patients included in the studies was 990.

**Conclusion:**

Myofibroblasts are a significant component in stroma of oral cancer cases, though not identified in all cases. This systematic review shows that clinical, pathological, and immunohistochemistry tests have correlated the presence of high myofibroblast count in oral cancer cell stroma.

**Key Messages:**

Myofibroblasts play a significant role in oral cancer invasion and progression. Various studies have demonstrated their association with oral cancer. This review tends to highlight their role in the pathogenesis of oral cancer over the decade.

**How to cite this article:**

Sekhon HK, Sircar K, Kaur G, Marwah M. Evaluation of Role of Myofibroblasts in Oral Cancer: A Systematic Review. Int J Clin Pediatr Dent 2016;9(3):233-239.

## INTRODUCTION

The myofibroblasts are a type of fibroblasts, which constitute a family of paracrine cells that play an important role in the regulation of fundamental processes, such as cell motility, proliferation, differentiation, apoptosis, morphogenesis, tissue repair, inflammation, and the immune response.^1-3^ It has been identified as the cell which produces stress fibers, have alpha-smooth muscle expression and even help in the production of tension, collagen fibrils, and growth factors.^4^ Their presence has been described practically in all fibrotic situations characterized by tissue retraction and remodeling.^5^ In many organs like liver, lung, and kidney, they are primarily involved in fibrosis. In the wound tissue, it is implicated in wound strengthening by extracellular collagen fiber deposition and then wound contraction by intracellular contraction and concomitant alignment of the collagen fibers by integrin-mediated pulling onto the collagen bundles.^6^ Myofibroblast may be considered as a foe or friend due to their beneficial role in normal healing granulation tissue and its damaging effects when it occurs in hypertrophic scars, scleroderma, dupuytrene disease, fibromatosis, fibrotic response to implants, lung fibrosis, heart fibrosis, kidney fibrosis, atheromatous plaque evolution, and chronic asthma.

The term “oral cancer” includes all the malignancies arising from lips, oral cavity, oropharynx, nasopharynx, hypopharynx, and other ill-defined sites within lip, oral cavity, and pharynx.^7^ In accordance to the study of diverse pathologic conditions in which the myofibroblast has been described, three fundamental processes were identified in 1980: Diverse responses to injury and repair phenomena, quasi-neoplastic proliferative conditions, the stromal response to certain forms of neoplasia.^8^ Many invasive and the metastatic carcinomas, especially those characterized by their hard consistency, retraction, and fixation to adjacent tissues, elicit a desmoplastic stromal reaction. The reaction that occurs in these carcinomas is brought about by stromal myofibroblasts.^9,10^

It was originally believed that the myofibroblastic stromal reaction represented a host response to cancer possibly to contain the invasive neoplasm.^11^ However, few authors have suggested that in invasive sarcomas, the stromal myofibroblast secretes stromal-degrading enzymes, which would favor cancer invasion.^12^ Myofibroblasts also produce a variety of factors that are involved in the pathogenesis of oral submucous fibrosis (OSMF).^13^ Myofibroblasts interact with epithelial cells and other connective tissue cells and may thus control phenomenon as tumor invasion and angiogenesis.^14^ Some studies have suggested secretion of enzymes that degrade the extracellular matrix, thereby facilitating tumor invasion by the myofibroblasts.^9^ The aim of this study was to systematically review the literature on the role of myofibroblasts in oral cancer development and progression.

## MATERIALS AND METHODS

The bibliographic searches were conducted in several electronic databases using all publications in PubMed, PubMed central, EMBASE, CancerLit, Google scholar, and Cochrane CCTR between 1990 and June 2015.

Criteria for considering studies:

Studies were considered eligible for study when they fulfilled the following criteria:

- Randomized controlled trials
- Experimental studies conducted on human species
- Observational studies with a comparison and control group.
- Full-text review articles
- Case reports

Exclusion criteria were as follows:

- Duplicate studies
- Unpublished data
- Studies involving animal species.

The primary outcome considered in this study was to evaluate the role of myofibroblasts in progression of oral cancer and analyze their prevalence in oral cancer cases. Other outcome included was prevalence in potentially premalignant oral lesions.

## SEARCH STRATEGY

Articles with relevance to search were identified from the abovementioned electronic databases. The search strategy used the terms from three categories: Oral anatomical parts, cancer, myofibroblasts, and was supplemented with search of text (Table 1).

In addition, hand searching was performed for full-text articles from the following journals: British Dental Journal, J Pathol, J Oral Pathol Med, Oral Oncology, and Histochem Cell Biol. For each included study, the following data were recorded: Year of publication, country of origin, details of participants, including demographic characteristic and details of study design according to Section 6.7.1 of Cochrane Reviewer’s Handbook.^15^

## RESULTS

The search of all publications from various electronic databases revealed 1,371 citations (Flow Chart 1).^16^ Searches of EMBASE, Google scholar, CancerLit, Cochrane CCTR, and bibliographies of review articles did not reveal any further relevant studies that had not been identified by PubMed search. Similarly, hand searching in the identified journals did not identify any other studies.

However, after implementing the terms of the search-strategy inclusion criteria, the studies that could be considered for systematic review reduced to 43 (Table 2).^1,2,6,9,10,11-14,17-48^

**Table 1:** **Table 1:** Keywords used in the literature search

Oral		Cancer		Myofibroblast	
Mouth		Mouth neoplasm			
Lip		Precancerous condition			
Gingiva		Tumor			
Tongue		Carcinoma			
Oropharynx		Malignant Dysplasia			

**Flow Chart 1:**
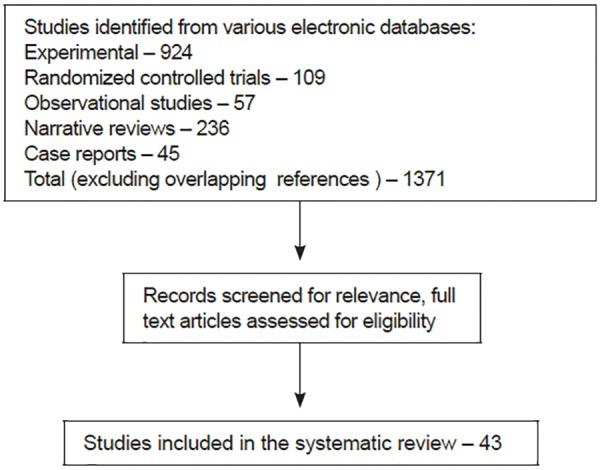
Study design

**Table 2:** **Table 2:** Characteristics of studies included in investigating prevalence of myofibroblasts in oral cancer

*References*		*Year of* *publication*		*Journal of* *publication*		*Details of the study*	
Zidar et al^17^		2002		Oncology		Sample - n_1_ = 42 (resected larynx) n_2_ = 40 (laryngeal biopsies of epithelial hyperplastic lesions and squamous carcinoma-myofibroblast found exclusively in squamous carcinoma)	
Barth et al^18^		2004		Virchows Arch		Sample - n = 39 (OSCC and tumor-free oral mucosa) 31 carcinomas showed increased stromal a SMA positive myofibroblast	
Lewis et al^19^		2004		Br J Cancer		Sample - n = 25 (15 OSCC and 10 fibro epithelial hyperplasia) Myofibroblast**s** differentiation is commonly seen in the stroma of OSCC (11), particularly at the invasive front of the tumor, no a SMA positive myofibroblast in the connective tissue of fibroepithelial hyperplasia	
Vered et al^20^		2005		Oral Oncol		Sample - n = 53, high frequency of stromal myofibroblast in known aggressive odontogenic lesion, such as odontogenic keratocyst parakeratinized type and solid ameloblastoma implies that myofibroblast can contribute to the biological behavior of these odontogenic lesions	
Kellermann et al^21^		2007		Histopathology		Sample - n = 117, myofibroblast in the stroma of the oral carcinoma are associated with poor prognosis	
Kellermann et al^22^		2008		Oral Oncol		60% of the OSCC contain myofibroblast in the stroma of the tumor During tumor invasion OSCC-derived TGF-p 1 promote fibroblast myofibroblast transdifferentiation and tumor cell proliferation can be induced by factors released from myofibroblast favoring tumor growth	
Verad et al^23^		2009		Cancer Microenviron		Sample - n = 19 (pair matched-oral tongue SCC and metastatic tumor to regional lymph node), expression of cancer-associated fibroblast is common to both primary and metastatic SCC	
Franz et al^24^		2009		Histochem Cell Biol		Sample - n = 152, Snail-positive cell in the stroma of OSCC invasive front without statistically significant correlation, histological grade, or nodal metastasis.	
Kawashiri et al^25^		2009		Head Neck		Sample - n = 84, high level of stromal collagen fibers in invasive tumors, myofibroblast appearance increased with increasing tumor invasiveness with frequent lymph node metastasis	
Etemad-Moghadam et al^26^		2009		J Oral Pathol Med		Sample - n = 70, presence of myofibroblast in the stroma of OSCC but not in dysplasia and normal mucosa	
Franz et al^27^		2010		J Oral Pathol Med		Sample - n = 5, mediated by myofibroblast OSCC development is associated with a stromal upregulation of laminin isoform possibly contributing to a migration promoting microenvironment	
Seifi et al^28^		2010		Asian Pac J Cancer Pre		v Sample - n = 54, increase in the number of myofibroblast and change in the distribution pattern occur during carcinogenesis signifying their role in tumor invasion characteristics	
Sobral et al^29^		2011		Oral Oncol		Sample - n = 30, myofibroblast in the stroma of OSCC may influence proliferation and invasion	
Salgueiredo-Giudice et al^30^		2011		Oral Surg Oral Med Oral Pathol Oral Radiol Endod		Sample - n = 3, demonstration of IHC profile of oral inflammatory myofibroblastic tumor along with morphological analysis reveals positive for calponin, vimentin, a-SMA, fibronectin	
Angadi et al^13^		2011		J Oral Pathol Med		Sample - n = 85, statistically significant increase in the myofibroblast between early and advance stages was observed	
Sridhara et al^31^		2013		J Oral Maxillofac Pathol		Sample - n = 10, a-SMA cases were more in the metastatic group than in the nonmetastatic tumor	
Lucio et al^32^		2013		Braz J Otorhinolaryngol		Myofibroblasts are important components of the stroma for SCC	
Angadi et al^33^		2014		Ada Odontol Scand		Sample - n = 65, (50-OSCC and histologically normal mucosa adjacent to OSCC, 15-control) significant co-relation was established for the presence of myofibroblast in the stroma of OSCC and HNMAOSCC. Myofibroblasts are early stromal change in the HNMAOSCC that highlights the possible role of myofibroblast as likely mediator for field cancerization	
Routray et al^34^		2014		Oral Dis		Myofibroblast can arise locally from endothelial mesenchymal transformation at the invasive edge of the cancer leading to development of high-grade malignancies and poor prognosis	
Pinisetti et al^35^		2014		J Oral Maxillofac Pathol		Myofibroblast in focal epithelial dysplasia and SCC revealed a higher number of myofibroblast in OSCC	
Rao et al^36^		2014		J Clin Diagn Res		Sample n = 62 (41 - OSMF, 10 - OSMF with dysplasia and 11 - OSCC). Presence of myofibroblasts was significantly higher in OSCC	
Luksic et al^49^		2015		Int J Oral Maxillofac Surg		Sample n = 152, myofibroblast proliferation was suggested to facilitate tumor invasion and distant metastasis	
Guan et al^50^		2015		Histopathology		Immunohistochemically, significant difference was observed in a-SMA expression in between normal controls and adenoid cystic carcinoma. This study demonstrated presence of myofibroblasts in adenoid cystic carcinoma.	
Jensen et al^51^		2015		J Oral Pathol Med		In this study, budding tumor cells had decreased expression of E-cadherin. Thus, it is suggested that budding tumor cells in OSCC is not dependent upon either myofibroblast or complete epithelial― mesenchymal transition.	

OSCC: Oral squamous cell carcinoma; HNMAOSCC: Histologically normal mucosa adjacent to oral squamous cell carcinoma; IHC: Immunohistochemistry; SMA: Smooth muscle actin; OSMF: Oral submucous fibrosis

## DISCUSSION

In neoplasia, proliferation of myofibroblasts was as a host stromal response to invasive carcinomas characterized by desmoplasia.^2^ The persistence of myofibroblast in a fibrotic lesion leads to scarring along with the functional impairment of the affected organ. The sustained myofibroblast activation stimulates the dysfunction repair mechanisms, leading to accumulation of fibrotic extracellular matrix, i.e., rich in collagens that bind to form fibrous bundles that are resistant to degradation. The fibrotic extracellular matrix disrupts cell polarity and stimulates cell proliferation, which creates an environment for the cancer formation and progression. Myofibroblast-induced inflammation and angiogenesis facilitate tumor growth and progression.^37^

Many epithelial tumors are characterized by the local accumulation of connective tissue cells and extracellular material; this phenomenon is known as the stromal reaction. The interaction of myofibroblast with epithelial cells and other connective tissue cells may control such phenomenon as tumor invasion and angiogenesis.^14^

In the recent history some studies have suggested secretion of enzymes that degrade the extracellular matrix, thereby facilitating tumor invasion by the myofibroblasts.^9^

Transdifferentiation of the fibroblasts to the myofibroblasts is a crucial and early event in tumorigenesis, which is mediated by the growth factors and cytokines, such as transforming growth factor-beta (TGF-beta) expressed by the tumor cells.^38^

Squamous carcinoma cells may induce a myofibroblast phenotype in primary fibroblasts through the secretion of TGF-beta.^39^ Transforming growth factor-beta causes cancer progression through paracrine and autocrine effects. Paracrine effects of TGF-beta include the stimulation of angiogenesis, escape from immuno-surveillance and recruitment of myofibroblasts, while the autocrine effects of TGF-beta in cancer cells with a functional TGF-beta receptor complex may be caused by a convergence between TGF-beta signaling and beta-catenin or activating Ras mutations.^40^

The myofibroblasts along with immune cells support blood vessel formation, breakdown of basement membrane barriers, and facilitate tumor invasion and metastasis. They significantly upregulate the secretion of hepatocyte growth factor, which promotes invasion of squamous cell carcinoma.^39^

Various experimental and clinical observations indicate the production of pro-invasive signals by the myofibroblasts which are implicated in cancer pain. N-Cadherin, expressed by myofibroblasts, promotes matrix invasion, perineural invasion, muscular invasion, and transendo-thelial migration.^40^

Epithelial cell proliferation is mediated by growth factors and inflammatory mediators secreted by myofibroblasts. The role of myofibroblasts in promoting invasion has been shown in numerous aggressive and malignant neoplasms. Zidar et al^17^ demonstrated that myofibroblasts were positive for vimentin and smooth muscle actin. This indicated that invasion beyond the basement membrane is necessary for the occurrence of the myofibroblastic stromal reaction. Two patterns of stromal reaction were observed in squamous carcinomas:

1. Characterized by a marked proliferation of myofi-broblasts and desmoplasia, with scarce lymphocytic infiltration. This pattern was associated with well- or moderately differentiated squamous carcinoma.
2. Characterized by few myofibroblasts, weak desmoplasia, and dense lymphocytic infiltration. This pattern was associated with moderately or poorly differentiated squamous carcinoma.

The degree of myofibroblast proliferation was inversely related to the density of lymphocytic infiltration.^17^ In invasive oral squamous cell carcinoma, an increase in smooth muscle actin-positive myofibroblasts has been observed. Etemad-Moghadam et al^26^ conducted a study that demonstrated an increased number of myofibroblasts in oral squamous cell carcinomas compared to normal and dysplastic epithelium.

Kellermann et al^21^ studied the prognostic significance of myofibroblasts in squamous cell carcinoma of tongue, preleukoplakia with histological dysplasia, and in normal tongue mucosa. No myofibroblasts were detected in the stroma of the normal mucosa or epithelial dysplasia which is in agreement with the results obtained by Etemad-Moghadam et al.^26^ Lewis et al^19^ demonstrated the presence of myofibroblast in the vicinity of invasive squamous cell carcinoma but not in the mucosal polyps. Kellermann et al^21^ and Vered et al^23^ have described the presence of the myofibroblast in the stroma of most human oral squamous cell carcinoma. Two dominant patterns discovered were:

1. Spindle
2. Network

In the “network” pattern, myofibroblasts are exceptionally abundant and occupy almost the entire tumor stroma.

The “spindle” pattern is characterized by stromal myofibroblast that have spindle-shaped morphology and are located at the periphery of carcinomas as one to three concentric layers, a pattern that can also be found adjacent to a few or many tumor islands/nests.^21,23^

The presence of myofibroblasts in squamous cell carcinoma is considered to be inductive phenomena. The epithelial-stromal interactions, different growth factors released by malignant epithelial cells induce, have been considered as source for the myofibroblasts.^26^

In a study to assess the frequency of stromal myofibroblast in the different odontogenic cysts and tumors, Vered et al^20^ found that the number of α smooth muscle-actin positive stromal cells or myofibroblasts was significantly higher in odontogenic keratocysts as compared to den-tigerous cysts. The myofibroblast counts in the parakera-tinized odontogenic cyst and solid ameloblastoma were not significantly different from that in the squamous cell carcinoma. The number of myofibroblasts in the unicystic ameloblastoma and the ameloblastic fibroma was comparatively lower than that in the more aggressive odon-togenic tumors and cysts. Thus, it was suggested that the myofibroblast has the potential to facilitate progression of epithelial lesions, and this can contribute to the biological behavior of these odontogenic lesions.

Based on semi-quantitative histological studies, myofibroblasts have been associated with tumor progression in various ways. Kellermann found an abundance of myofibroblast to be associated with the N-stage but not with tumor size (T stage).^21,41-48^ Most studies show increased quantities of myofibroblast to be associated with poor prognosis.

## CONCLUSION

Myofibroblasts are significant components in the stroma of oral cancer lesions, though they may not be identified in all cases. A review of the literature indicates that myofibroblasts play an important role in facilitating invasion by oral squamous cell carcinoma by expression of growth factors, cytokines, extracellular components,^21^ and various proteolytic enzymes. This systematic review shows that clinical, pathological, and immunohistochemistry tests have correlated the presence of high myofibroblast count in oral cancer cell stroma. Most of these observations have been made in the last few years from 1990 to 2015. Thus there is a need for further research to understand the molecular mechanisms by which myofibroblasts impact the biological behavior of oral squamous cell carcinoma.

## SUMMARY OF WORK DONE BY THE CONTRIBUTORS

All the authors have worked cooperatively on the study. The design of the study was well-versed among all the authors and valuable suggestions were discussed and implemented. All authors coordinated well for the manuscript literature search, preparation, editing, and corrections to be made after review.
